# Synthesis and evaluation of an ^18^F‐labeled derivative of F3 for targeting surface‐expressed nucleolin in cancer and tumor endothelial cells

**DOI:** 10.1002/jlcr.3439

**Published:** 2016-09-04

**Authors:** Phoebe Y.H. Lam, Christopher R.T. Hillyar, Sarah Able, Katherine A. Vallis

**Affiliations:** ^1^ Department of Oncology, CR‐UK/MRC Oxford Institute for Radiation OncologyUniversity of OxfordUK

**Keywords:** [^18^F]Al‐F, endothelial cells, F3, nucleolin receptor, transglutaminase

## Abstract

The surface overexpression of nucleolin provides an anchor for the specific attachment of biomolecules to cancer and angiogenic endothelial cells. The peptide F3 is a high‐affinity ligand of the nucleolin receptor (NR) that has been investigated as a carrier to deliver biologically active molecules to tumors for both therapeutic and imaging applications. A site‐specific PEGylated F3 derivative was radiolabeled with [^18^F]Al‐F. The binding affinity and cellular distribution of the compound was assessed in tumor (H2N) and tumor endothelial (2H‐11) cells. Specific uptake via the NR was demonstrated by the siRNA knockdown of nucleolin in both cell lines. The partition and the plasma stability of the compound were assessed at 37°C. The enzyme‐mediated site‐specific modification of F3 to give NODA‐PEG‐F3 (NP‐F3) was achieved. Radiolabeling with [^18^F]Al‐F gave ^18^F‐NP‐F3. ^18^F‐NP‐F3 demonstrated high affinity for cancer and tumor endothelial cells. The siRNA knockdown of nucleolin resulted in a binding affinity reduction of 50% to 60%, confirming cell surface binding via the NR. NP‐F3 was stable in serum for 2 h. ^18^F‐NP‐F3 is reported as the first ^18^F‐labeled F3 derivative. It was obtained in a site‐specific, high‐yield, and efficient manner and binds to surface NR in the low nanomolar range, suggesting it has potential as a tumor and angiogenesis tracer.

## 1 Introduction

Nucleolin is a nonribosomal nucleolar protein consisting of 710 amino acids. Nucleolin is the predominant protein constituent of the nucleolus and is also expressed at the cell surface after the induction of nucleolin mRNA.^1^, ^2^ Nucleolin does not possess a hydrophobic domain for association with the plasma membrane; however, nucleolin clustering, which is dependent on the intracellular actin cytoskeleton, does occur on the external aspect of the membrane. The induction of surface nucleolin occurs independently of nuclear nucleolin and has been linked to the proliferative capacity of normal and tumor cells. Surface nucleolin is self‐cleaved in normal cells, autocatalyzing its own degradation. By contrast, constantly induced nucleolin mRNA in tumor cells results in the synthesis of nucleolin that rapidly translocates to the cell surface where it serves as a receptor for several ligands. The half‐life of cell surface nucleolin is estimated to be approximately 1 h, whereas that of nuclear nucleolin is greater than 8 h.^3^, ^4^ Cell surface nucleolin expression is elevated in rapidly proliferating cells,^1^, ^5^ and the overexpression of cell surface nucleolin receptor (NR) has been observed in tumor endothelial cells^6^ and cell lines derived from human cancers of breast,^7^ gastric,^8^ colonic,^9^ and lung^10^ origin. In human gliomas, an increase in cell surface NR expression has been associated with histological grade.^11^

The overexpression of the NR in rapidly proliferating cells such as tumor cells and tumor endothelium can be exploited for specific cell targeting. Cell surface NR serves as a target for endostatin^6^ and various carcinogenic ligands.^5^, ^8^, ^12^ The tumor‐homing peptide F3 (KDEPQRRSARLSAKPAPPKPEPKPKKAPAKK) is a 31 amino acid synthetic fragment of the human high mobility group protein 2 (HMGN2).^13^ F3 was discovered using phage display libraries while searching for proteins capable of homing to the tumor and its vasculature, and it has since been used as a payload carrier to deliver biologically active molecules to tumors. For example, F3‐functionalized nanoparticles that target the blood vessels of various tumor xenografts^14^, ^15^, ^16^, ^17^, ^18^, ^19^ and F3 conjugates that deliver siRNA and oligonucleotides into cells have been reported.^14^, ^17^, ^20^ In nuclear medicine, various F3 peptide derivatives have been synthesized for SPECT imaging as well as α‐particle and Auger electron therapy. F3 peptide with a C‐terminal cysteine residue radiolabeled with iodine‐125 showed high specific uptake in tumor cell lines but failed to accumulate in the tumor in vivo.^21^ In another study, the therapeutic efficacy of F3 radiolabeled with the alpha‐emitting isotopes bismuth‐213 and actinium‐225 were compared alongside each other in a preclinical mouse model of peritoneal carcinomatosis.^22^ Both compounds showed similar antitumor efficacy. We previously reported on the indium‐111‐labeled derivative ^111^In‐BnDTPA‐F3, which homed to the nucleus and, in particular, the nucleoli of tumor cells via cell surface NR. ^111^In‐BnDTPA‐F3 reduced clonogenic survival in vitro and caused tumor growth inhibition in vivo.^23^

In view of the tumor‐homing and inhibitory properties of ^111^In‐BnDTPA‐F3, it was of interest to develop an alternative radiotracer labeled with fluorine‐18 (^18^F) as a potential positron emission tomography (PET) tracer. Fluorine‐18 is widely used for clinical PET imaging owing to its half‐life of 109.8 min, which is brief enough to negate the need for prolonged radiation protection measures but long enough to allow complex radiosynthesis and imaging of slow metabolic processes. The weak positron energy (635 keV) and the short positron range (2.3 mm) of ^18^F in matter account for the favorable spatial resolution it provides using modern PET cameras.^24^ ^18^F is commonly attached to a carbon atom on the carrier biomolecule or a prosthetic group subsequently coupled to the biomolecule. Recent advances in ^18^F radiochemistry include the development of ^18^F‐bond formation with silicon and boron.^25^, ^26^ Efforts to increase ^18^F radiochemical yield and to minimize reaction times are frequently reported in the literature, with McBride et al. receiving attention for the description of radiofluorination using an aluminum‐fluoride complex.^27^ The Al‐F bond is the strongest of fluoride‐metal bonds, with (AlF)^2+^ forming stable complexes with chelators.^28^ This strategy allows for the incorporation of ^18^F into biologically active ligands with low solubility in organic solvents. Peptides and small molecules have been radiolabeled efficiently using this approach, with a drastically reduced synthesis time and increased radiochemical yields, potentially facilitating easy transition to automated radiosynthesis in a clinical setting.^29^, ^30^

Integrin αvβ3 expression has been studied extensively as a biomarker of both angiogenesis and metastasis and visualized using radiolabeled monomeric or multimeric RGD (Arg‐Gly‐Asp) peptides and nanoparticles. However, the hepatobiliary elimination of RGD peptides leads to liver accumulation and slow excretion from the large intestine.^31^ By contrast, the positively charged F3 peptide is more hydrophilic and is therefore expected to undergo rapid renal excretion, decreasing the background signal in the abdominal region.

We herein report the first ^18^F‐labeled F3 derivative known to date. F3 peptide was PEGylated to increase its biological half‐life, and this conjugation was conducted in a site‐specific manner. The modified peptide was radiolabeled with an [^18^F]AlF‐chelator complex to give ^18^F‐NP‐F3, which was shown to bind cancer and tumor endothelial cells via the NR.

## 2 Experimental

### 2.1 Materials

All chemicals and solvents were obtained at analytical grade from Sigma‐Aldrich (Dorset, UK), Merck (Hertfordshire, UK), CheMatech (Dijon, France), or Macrocyclics (Dallas, TX) and were used without further purification. F3 peptide (KDEPQRRSARLSAKPAPPKPEPKPKKAPAKK, MW = 3432 g/mol) was obtained from Cambridge Peptides (Cambridge, UK). Size and purity were confirmed by reverse phase high‐performance liquid chromatography (HPLC) and mass spectroscopy. Bacterial transglutaminase was obtained from Zedira (Darmstadt, Germany). No‐carrier‐added (NCA) fluorine‐18 dissolved in [^18^O]H_2_O was provided by PETNET Solutions (Middlesex, UK). [^111^In]InCl_3_ was provided by Perkin Elmer (Waltham, MA). Antibodies against nucleolin (ab129200), β‐actin (ab8227), H2AZ (ab4174), β‐integrin (ab179471), and α‐tubulin (ab176560) were obtained from Abcam (Cambridge, UK); anti‐PARP‐1 antibody F‐2 was obtained from Santa Cruz Biotechnology, Inc. (Dallas, TX); and all other antibodies were obtained from Invitrogen (Paisley, UK).

### 2.2 Synthesis of ^18^F‐NP‐F3

*p*‐SCN‐Bn‐NOTA (0.11 mmol) in anhydrous DMSO (1 mL) was added to a mixture of tert‐butyloxycarbonyl (BOC)‐PEG‐NH_2_ (1 Eq) and N,N‐diisopropylethylamine (DIPEA) (1 Eq) in anhydrous dimethylsulfoxide (DMSO) (1 mL) in a dried flask. The reaction was carried out overnight at room temperature and purified by column chromatography (pentane/ethyl acetate, 4:1). The product was redissolved in trifluoroacetic acid (TFA)/CH_2_Cl_2_ (1:1) (2 mL) and stirred at room temperature for 1 h until complete BOC cleavage had occurred to give NODA‐PEG‐NH_2_. F3 peptide (0.32 mmol), NODA‐PEG‐NH_2_ (1 Eq), and bacterial transglutaminase (1 U/mL) were dissolved in potassium‐free phosphate buffered saline (PBS) buffer adjusted to pH 8 with a saturated aqueous solution of Na_3_PO_4._ The reaction mixture was incubated at 37°C until steady‐state conditions were achieved. Excess enzyme and chelator were removed using centrifugation dialysis (3 K). NCA fluorine‐18 (1 GBq) was trapped on an anion‐exchange cartridge (Sep‐Pak Light Accell Plus QMA, Waters AG, Milford, MA) preconditioned with 0.5 M K_2_CO_3_ (5 mL) and water (5 mL). The NCA ^18^F‐fluoride was washed with metal‐free water and eluted with 0.4 M KHCO_3_ (0.5 mL) and subsequently adjusted to pH 4 with metal‐free glacial acetic acid. A solution of 2 mM AlCl_3_ (0.25 mL) in 0.1 M sodium acetate buffer (pH 4) and NODA‐PEG‐NH_2_ were added sequentially and stirred at 100°C for 20 min. After dilution with metal‐free water, the reaction mixture was purified by reversed phase solid phase extraction (SPE) cartridge (Sep‐Pak light C18, Waters AG), and the product was eluted by EtOH (0.5 mL) and formulated in PBS/EtOH (95:5). For pharmacological comparisons, ^111^In‐BnDTPA‐F3 was synthesized as previously described.^23^

### 2.3 Cell culture

The 231‐H2N variant of the human breast cancer cell line, MDA‐MB‐231 (referred to as “H2N”), was a gift of Dr. Robert Kerbel (Sunnybrook Health Sciences Centre, Toronto). The SV40‐transformed human tumor endothelial cell line, 2H‐11, was obtained from the American Type Culture Collection (ATCC, Manassas, VA, USA). The cells were cultured in Dulbecco's modified Eagle's medium (Sigma‐Aldrich) supplemented with 10% fetal bovine serum (Invitrogen), 2 mM gluatmine (Sigma‐Aldrich) and 100 U/mL penicillin/streptomycin (Invitrogen) at 37°C in 5% CO_2_.

### 2.4 Cell binding assays

For saturation binding determination, a suspension of H2N or 2H‐11 cells (7000 cells, 200 μL) was added to Eppendorf tubes cooled in ice, followed by the radiotracer (200 μL) in varying concentrations. Triplicates of each substrate concentration were investigated. The cell suspensions were incubated at 4°C for 40 min and then centrifuged (800 g, 4°C, 10 min). The supernatant was decanted, and excess radioligand was removed. The cells were lysed with 0.1 M NaOH (500 μL) and transferred into counting tubes, and the amount of radioactivity was measured using a γ‐counter (Wizard, Perkin Elmer). Competitive inhibition was determined by adding a suspension of H2N or 2H‐11 cells (8000 cells, 225 μL) to Eppendorf tubes containing F3 in varying concentrations (250 μL) and ^111^In‐BnDTPA‐F3 (25 μL, 20 nM/well) in triplicates. The cells were incubated at 4°C for 45 min and centrifuged (800 g, 4°C, 10 min); the supernatant was decanted, and excess radioligand was removed. The cells were lysed with 0.1 M NaOH (500 μL) and transferred to counting tubes, where radioactivity was measured using a γ‐counter. siRNA (Thermoscientific, UK) directed against nucleolin was resuspended in RNase‐free buffered solution and diluted to 100 μM and mixed at room temperature or 37°C for 90 min. The siRNA solution was further diluted in serum‐free medium to give a final working concentration of 1 μM siRNA per well in a 96‐well plate. H2N or 2H‐11 cells in culture were trypsinized and resuspended in serum‐free medium, diluted to a plating density of 20% to 30% and added to a 96‐well plate (100 μL per well). The cells were incubated at 37°C overnight. Medium was removed from the cells, and the siRNA delivery mix (100 μL) was added to triplicate wells. The cells were incubated at 37°C for 72 h and assessed for protein knockdown. The radiotracer in various concentrations (50 μL) was added in triplicate to the knockdown cells in 96‐well plates and incubated at 4°C for 2 h. Nonspecific binding was determined by competition with cold F3 (200 nM, 10 μL). The supernatant was removed after incubation, and the cells were washed with PBS (2 × 100 μL), lysed with 0.1 M NaOH (200 μL), and radioactivity measured using a γ‐counter.

### 2.5 Distribution coefficient determination

#### 2.5.1 UV detection

F3 in varying concentration and volume was added to Eppendorf tubes containing octanol saturated with Sørensen buffer (300 μL, pH 7.4) and Sørensen buffer (pH 7.4) saturated with octanol (300 μL) in triplicate. The tubes were gently and continuously mixed overnight at room temperature, after which the aqueous and organic phases of each tube were carefully isolated. Octanol was removed under vacuum overnight, and the residue was redissolved in water. The samples were analyzed by LC/MS (Waters; acetonitrile (ACN) w/0.1% TFA in H_2_O w/0.1% TFA, 2.5% to 40% over 7 min, 0.3 mL/min). The area under the curve of the peaks representing each phase was determined, and the experimental LogD value was derived from the formula:

$${\text{LogD}}_{\text{exptl}}=\mathrm{Log}\left[{\mathrm{CPM}}_{\mathrm{avg}}\left(\text{octanol phase}\right){/\mathrm{CPM}}_{\mathrm{avg}}\left(\text{aqueous phase}\right)\right]$$

#### 2.5.2 Detection by radioactivity

The radiotracer (1 MBq, 100 μL) was added to Eppendorf tubes containing octanol saturated with Sørensen buffer (200 μL, pH 7.4) and Sørensen buffer (pH 7.4) saturated with octanol (200 μL) in triplicates. The tubes were gently and continuously mixed over 1 h at room temperature, after which the aqueous and organic phases were carefully isolated, and two 60‐μL aliquots of each of the sample were placed in the γ‐counter for radioactivity determination. The experimental LogD was calculated from the above formula.

### 2.6 Serum stability study

The HPLC purified radiotracer (1 MBq in 10 μL) was added to bovine serum albumin solution (450 μL) and incubated at 37°C while mixing. At 0, 15, 30, 60, or 90 min, the mixture was quenched with ACN (150 μL) and centrifuged (500 g, 37°C, 5 min). The supernatant was removed and assessed by HPLC.

### 2.7 Cell internalization and retention assay

The radiotracer (550‐700 kBq per tube) was added to H2N or 2H‐11 cells (7000 cells/tube) and incubated at 37°C for 0, 5, 15, 30, 60, and 120 min. Glycine (0.1 M; pH 2.5, 500 μL) was added to the tubes and incubated at 4°C for 10 min. The tubes were centrifuged and the supernatant removed. The pellet was washed with PBS (500 μL), and the combined supernatant fractions were assessed using a γ‐counter to determine the radioactivity of the membrane‐bound fraction. The remaining pellet was lysed with 0.1 M NaOH, and the lysates were transferred to counting tubes to determine the radioactivity of the internalized fraction using a γ‐counter. To determine the extent of cellular retention, H2N or 2H‐11 cells were seeded overnight in 12‐well plates. Cell culture medium was replaced (1 mL/well), and the radiotracer was added to give a resulting concentration of 800 nM/well. The cells were incubated at 37°C for 2 h, after which the medium was removed, cells were washed with PBS (1 mL/well), and fresh medium was added (1 mL/well). At each time point (0, 5, 15, and 30 min; 1, 2, 4, 8, 12, and 24 h), medium was removed, the cells were washed thrice with PBS (1 mL/well), lysed with 1 M NaOH (1 mL/well), and the radioactivity of the retained tracer was determined using a γ‐counter.

### 2.8 Western blot analysis

H2N or 2H‐11 cells were separated into membranous, cytoplasmic, and nuclear fractions using a commercially available cell fractionation kit (Thermoscientific, UK). The protein content of each fraction was determined using a Pierce BSA protein assay kit (Thermoscientific, UK). Equal amounts of protein (15 μg) were subjected to electrophoresis on SDS‐PAGE and transferred to 0.45 μm nitrocellulose membranes (BioRad). The blotted membranes were immunostained with antinucleolin antibody (ab129200), counterstained with HRP‐conjugated IgG, and visualized by chemiluminescence (ECL). The relative amounts of total nucleolin expressed in each cell fraction were calculated by measuring the intensity of the bands using ImageJ.

## 3 Results and discussion

### 3.1 Competition binding of F3

**Figure 1:**
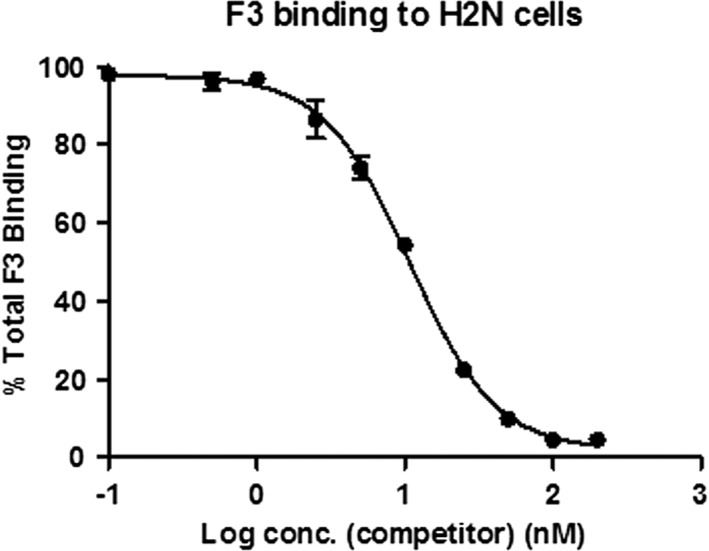
Competition binding of F3. F3 peptide was serially diluted and competed against ^111^In‐BnDTPA‐F3. The IC_50_ values were calculated to be 10.98 and 12.71 nM for H2N and 2H‐11 cells, respectively. The competition binding curve for 2H‐11 cells is shown in Supplementary Information

The human breast cancer cell line, MDA‐MB‐231/H2N (H2N), and the SV40‐transformed endothelial cell line, 2H‐11, are known to overexpress nucleolin at the cell surface and were used as representative cell lines for all the experiments reported in this article. To produce useful F3 derivatives for tumor targeting, it is important to first understand the pharmacological profile of the native peptide itself. In competition binding experiments, the IC_50_ values for F3 in H2N and 2H‐11 cells were 10.98 nM and 12.71 nM, respectively. These values served as a guideline for F3‐based compounds, in that any modification of the peptide should retain binding affinity in the low nanomolar range (Figure 1 and Supplementary Information). Nonspecific binding was found to be minimal for both cell lines.

### 3.2 Expression of nucleolin in breast cancer and endothelial cells

**Figure 2:**
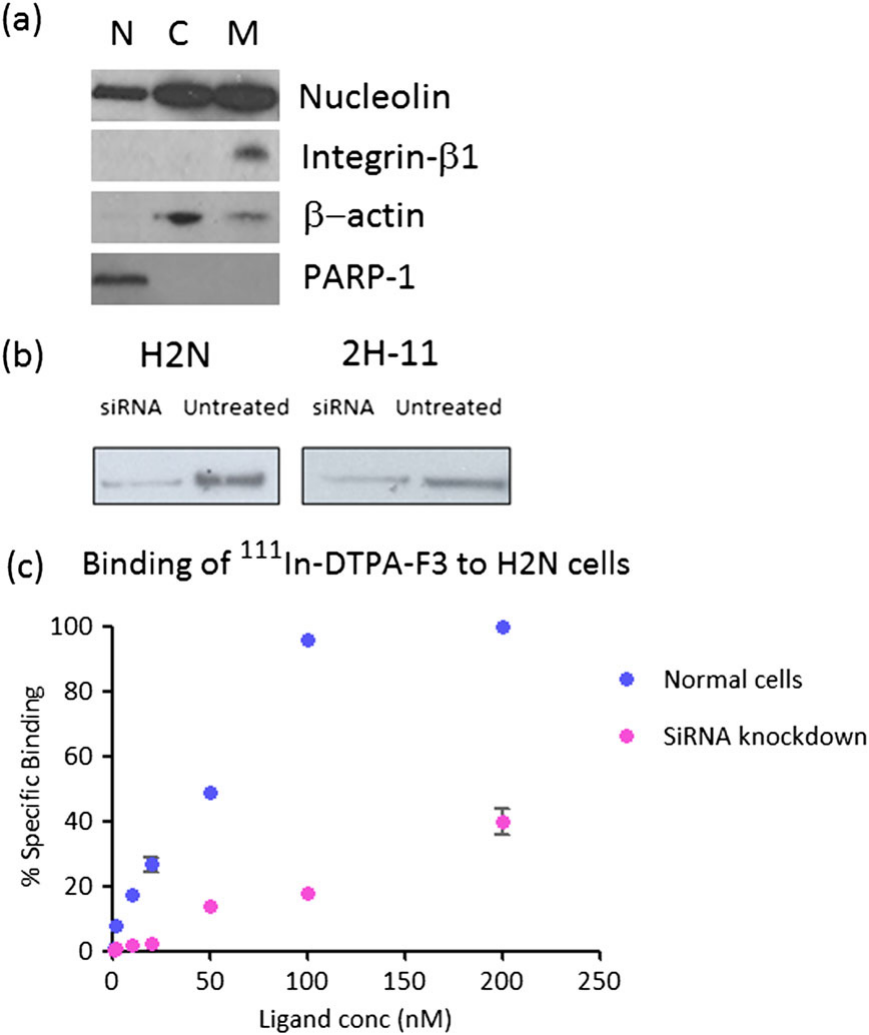
(A) Expression of nucleolin in nuclear (N), cytoplasmic (C), and membranous (M) fractions of H2N cells. Integrin‐β1, β‐actin, and PARP‐1 were used as membrane, cytoplasm, and nuclear markers, respectively. The nucleolin expression for 2H‐11 cells is shown in Supplementary Information. (B) Immunoblotting of nucleolin revealed the partial knockdown of nucleolin in siRNA‐treated cells. (C) Saturation binding curve of ^111^In‐BnDTPA‐F3 for normal H2N cells (in blue) and H2N cells treated with siRNA against nucleolin (in pink). Reduction in the amount of nucleolin accounted for approximately 60% loss of binding affinity. The same experiment was repeated for 2H‐11 cells, which demonstrated a 35% reduction in binding affinity of the radioligand in treated cells (see Supplementary Information)

Aliquots of both cell lines were fractionated into membrane, cytoplasmic, and nuclear fractions and Western blotting for nucleolin was performed. All 3 fractions for both cell lines showed a band at around 100 kDa, indicative of nucleolin, which has molecular weight of 106 kDa (Figure 2A and Supplementary Information).

### 3.3 Specific binding of F3‐based probes to the NR

To confirm that F3‐based compounds bind specifically to the NR on the cell surface of H2N and 2H‐11 cells, the siRNA knockdown of nucleolin in both cell lines was carried out. After siRNA transduction, Western blot analysis revealed a reduction in the amount of nucleolin in the knockdown cells (Figure 2B). Binding assays with ^111^In‐BnDTPA‐F3 in the untreated and siRNA‐treated lines were carried out. The maximum binding of ^111^In‐BnDTPA‐F3 was reduced by 60% and 35% in the siRNA‐treated versus untreated H2N and 2H‐11 cells, respectively (Figure 2C and Supplementary Information). This result indicates that the NR is responsible for F3 binding to H2N and 2H‐11 cells.

### 3.4 Retention of ^111^In‐BnDTPA‐F3 in cells

**Figure 3:**
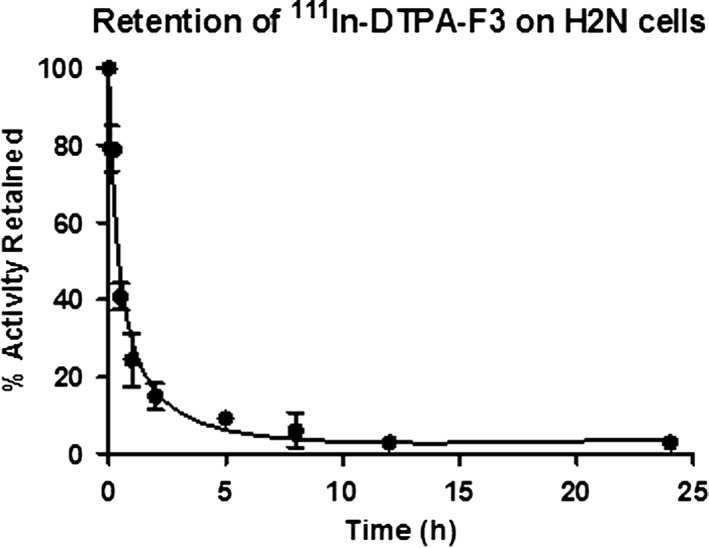
Retention of ^111^In‐BnDTPA‐F3 in H2N cells; 79% of radioactivity remained bound to the cells after incubation at 37°C for 5 min, and 24% remained bound at 1 h

^111^In‐BnDTPA‐F3 was reported previously to be taken up into the nucleoli of tumor cells via cell surface NR, causing decreased clonogenic survival in vitro and tumor growth inhibition in vivo.^23^ However, a rapid clearance of the compound was observed, as demonstrated by SPECT imaging and the biodistribution profile, which showed a high uptake in the kidneys (7% ID/g) and a low tumor uptake of 1% ID/g at 3 h post injection. ^111^In‐BnDTPA‐F3 was prepared as previously described.^23^ The *K*_d_ value of ^111^In‐BnDTPA‐F3 for H2N and 2H‐11 cells was determined to be 24.2 ± 3.05 nM (nonspecific binding NSB = 4.1%) and 25.6 ± 2.82 nM (nonspecific binding = 4.7%), respectively. The binding affinity of ^111^In‐BnDTPA‐F3 was, therefore, favorable, and 79% of the bound activity was retained after 5 min of incubation at 37°C (Figure 3).

### 3.5 Lipophilicity of F3 and ^111^In‐BnDTPA‐F3

The distribution of F3 between equal volumes of organic and aqueous phases was determined by HPLC, whereas that of radiolabeled ligands was indicated by radioactivity present in the aqueous and organic phases. F3 was found to be moderately hydrophilic, having an experimental LogD value of −3.47 ± 0.30. This result was expected, given that the peptide is rich in basic amino acids and carries an overall positive charge. The LogD for ^111^In‐BnDTPA‐F3 was −3.48; thus, in terms of structure and overall hydrophilicity, ^111^In‐BnDTPA‐F3 was similar to F3. This low LogD value may account for the rapid renal excretion of ^111^In‐BnDTPA‐F3 in vivo.^23^ As rapid renal metabolism would limit the bioavailability of the compound in vivo, we sought to increase the lipophilicity of a fluorine‐18‐labeled variant compound. A common way of prolonging biological half‐life is by linking peptides to 1 or more polyethylene glycol (PEG) chains. The PEG chain then also serves as a linker between the metal‐chelator and the peptide.

### 3.6 Synthesis of NODA‐PEG‐NH‐F3

**Figure 4:**
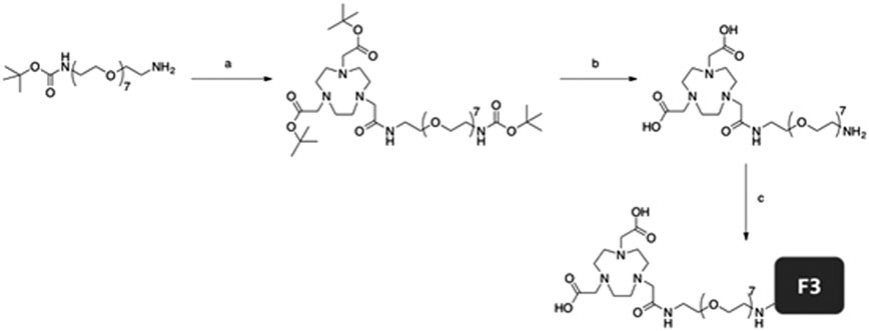
Synthesis of NODA‐PEG‐NH‐F3 (NP‐F3) (a) NOTA(tBu)_2_, HOBt, anhydrous DMF. (b) TFA/CH_2_Cl_2_. (c) F3, Bac. Tgase, pH 7.0.

Triazacyclononane derivatives such as NOTA and NODA are able to form stable complexes with aluminum fluoride in the form of (AlF)^2+^. X‐ray crystallography has previously shown that coordination of an aluminum atom to NOTA complexes results in a distorted geometry because of the restricted fixation of the chelating ligands. The extra carbonyl group on NOTA causes a 5‐membered ring to form after complexing with aluminum, which interferes with ^18^F binding and results in a much lower radiochemical yield.^32^, ^33^ Therefore, NODA is preferred over NOTA as the chelator for [^18^F]AlF radiolabeling. Synthesis began with the reaction of *p*‐SCN‐Bn‐NOTA with BOC‐PEG‐NH_2_. Subsequent BOC cleavage gave NODA‐PEG‐NH_2_ as the final product. We performed site‐specific conjugation of NODA‐PEG‐NH_2_ to F3 using enzymatic catalysis. Bacterial transglutaminase catalyses the peptide bond formation between glutamine and lysine residues, enabling site‐specific modification of peptides.^34^ As F3 contains only a single glutamine residue and chelator‐PEG‐NH_2_ is lysine mimicking, site‐specific PEGylation can be carried out without the need for additional functional groups to be added to the peptide. The reaction of NODA‐PEG‐NH_2_ with F3 in the presence of bacterial transglutaminase facilitated the conjugation of NODA‐PEG‐NH_2_ onto the single glutamine residue on F3, giving NODA‐PEG‐NH‐F3 (NP‐F3) as a homogeneous species with a chelator‐to‐peptide ratio of 1:1. The 4‐step reaction afforded NP‐F3 in 68% overall yield (Figure 4). It is important to note that the number of chelators per peptide in this case is an absolute value and not an average estimate. Site‐specific conjugation afforded a single species to bind to the target, allowing for the possibility of structure‐activity relationships to be established. NP‐F3 was then radiolabeled with [^18^F]AlF to give ^18^F‐NP‐F3. Radiosynthesis was highly dependent on the pH of the reaction environment, with pH 4 being optimal. Radiolabeling with [^18^F]AlF at 100°C for 20 min at pH 4 resulted in ^18^F‐NP‐F3 with a radiochemical yield of 78%.

### 3.7 Binding affinity, cellular internalization, retention, stability, and lipophilicity of NP‐F3

**Figure 5:**
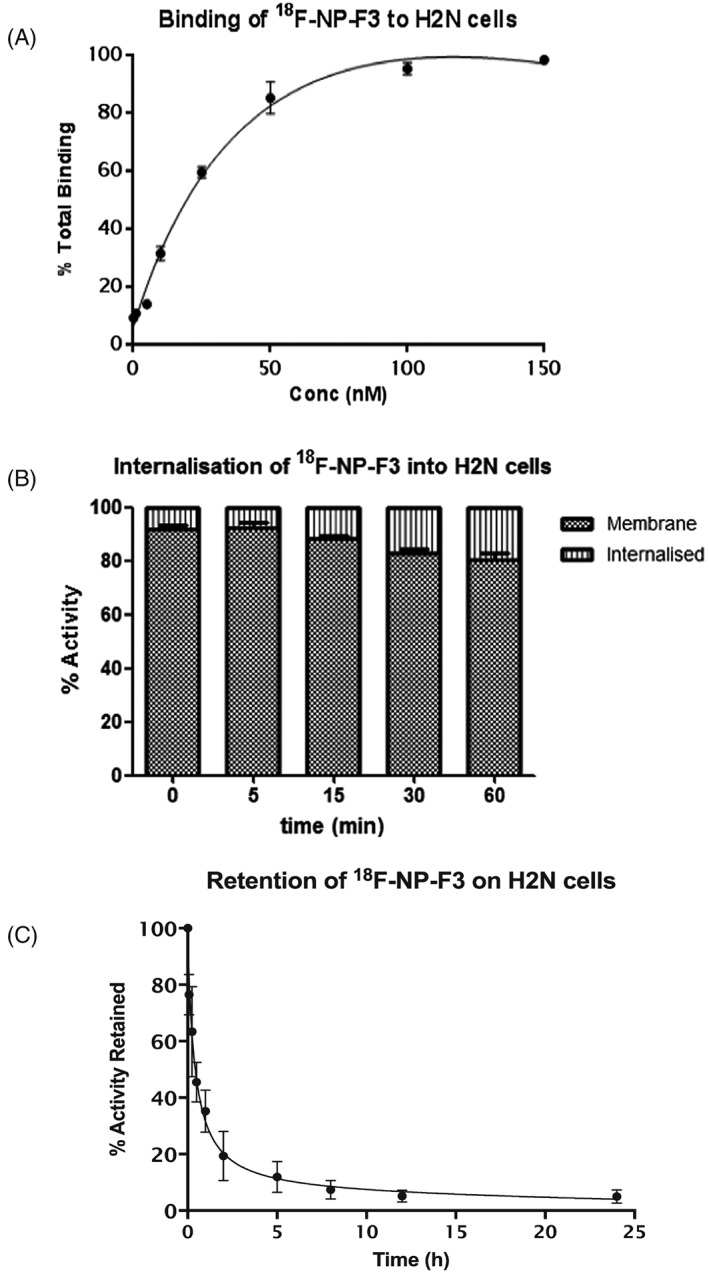
(A) Saturation binding curve of ^18^F‐NP‐F3 on H2N cells. ^18^F‐NP‐F3 was added to H2N cells in varying concentrations. The *K*_d_ value was found to be 46 nM for H2N, indicating that the compound retained specific affinity for the NR despite peptide modifications. The binding curve for 2H‐11 cells is presented in the Supplementary Information. (B) Intracellular distribution of ^18^F‐NP‐F3 in H2N cells. (C) Retention of ^18^F‐NP‐F3 on H2N cells; 34.2% of radioactivity remained bound to the cells after 1 h

^18^F‐NP‐F3 was tested in binding affinity assays with H2N and 2H‐11 cells, revealing *K*_d_ values of 46.38 ± 2.7 and 49.22 ± 4.1 nM respectively, which was found to be comparable with that of native F3 and ^111^In‐BnDTPA‐F3 (Figure 5A and Supplementary Information). Nonspecific binding was minimal in both cases (2.3% for H2N cells and 3.9% for 2H‐11 cells). Incubation of the radioligand with H2N and 2H‐11 cells at 37°C showed at least 80% accumulation of the tracer on the cell membrane (Figure 5B). The distribution was found to be stable over an hour. After 1 h, 34.2% of total uptaken activity remained bound to the cells (Figure 5C). To test its stability, ^18^F‐NP‐F3 was mixed with serum at 37°C and found to be stable for up to 2 h. The partition of ^18^F‐NP‐F3 between equal volumes of organic and aqueous phases gave an experimental LogD of −3.10 ± 0.08. As intended, therefore, ^18^F‐NP‐F3 was more lipophilic than F3 and ^111^In‐BnDTPA‐F3, and this would be predicted to lead to greater biological retention.

## 4 Conclusion

^18^F‐NP‐F3 is presented as the first fluorine‐18‐labeled F3 derivative for targeting the NR in tumors and the tumor endothelium. It binds to tumor cells and tumor endothelial cells with high affinity. The stability of ^18^F‐NP‐F3 and its affinity for the NR suggest it has potential as a tumor and angiogenesis tracer.

## Supporting information

Supplementary information to Figure 1. Competitive inhibition of F3. F3 peptide of varying concentration was competed against ^111^In‐BnDTPA‐F3. The IC_50_ value was calculated to be 12.71 nM for 2H‐11 cells.
Supplementary information to Figure 2a. Expression of nucleolin in cytoplasmic (C), membranous (M), and nuclear (N) fractions of 2H‐11 cells. The relative nucleolin expression in the 3 fractions is shown. Integrin‐β1, α‐tubulin, and histone H2 were used as membrane, cytoplasm, and nuclear markers, respectively.
Supplementary information to Figure 2c. Saturation binding curve of ^111^In‐BnDTPA‐F3 on normal 2H‐11 cells (in blue) and 2H‐11 cells treated with siRNA against nucleolin (in pink). Reduction of nucleolin accounted for approximately 35% loss in binding affinity. These results confirm that the radioligand is bound to tumor endothelial cells via the nucleolin receptor (NR).
Supplementary information to Figure 5a. Saturation binding curve of ^18^F‐NP‐F3 on 2H‐11 cells. ^18^F‐NP‐F3 was added to 2H‐11 cells in varying concentrations. The *K*_d_ value was found to be 49 nM for 2H‐11 cells, indicating that the compound retained specific affinity for the NR despite peptide modifications.

Supporting info item
