# Supplementary material for: Synthesis and evaluation of an 18F‐labeled derivative of F3 for targeting surface‐expressed nucleolin in cancer and tumor endothelial cells
Source: J Labelled Comp Radiopharm. 2016 Sep 4;59(12):492–9. doi: 10.1002/jlcr.3439 (PMC5082555; doi:10.1002/jlcr.3439)
Supplement: Supplementary file 1 — Supporting info item [file JLCR-59-492-s001.docx]

**Supplementary Information:**


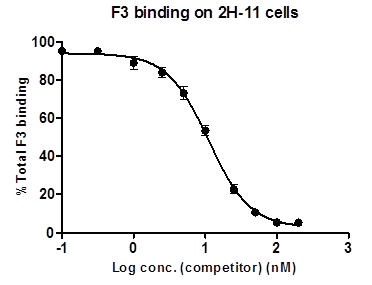


**Supplementary information to Figure. 1:** **Competitive inhibition of F3.** F3 peptide of varying concentration was competed against ^111^In-BnDTPA-F3. The IC_50_ value was calculated to be 12.71 nM for 2H-11 cells.


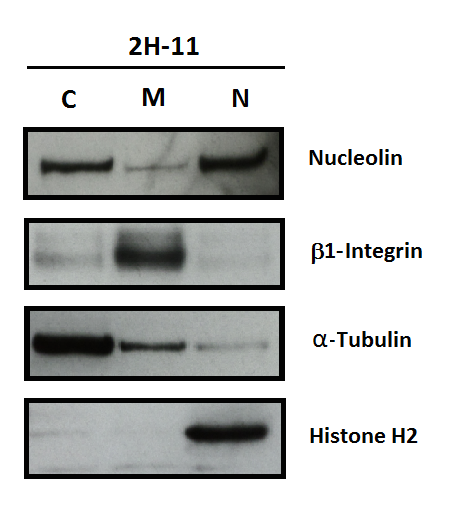


**Supplementary Information to Figure 2a. Expression of nucleolin in cytoplasmic (C) membrananous (M) and nuclear (N) fractions of 2H-11 cells.** The relative nucleolin expression in the 3 fractions is shown. Integrin-β1, α-tubulin and histone H2 were used as membrane, cytoplasm, and nuclear markers, respectively.


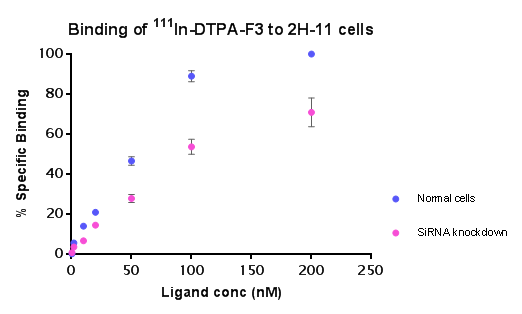


**Supplementary information to Figure. 2c** Saturation binding curve of ^111^In-BnDTPA-F3 on normal 2H-11 cells (in blue) and 2H-11 cells treated with siRNA against nucleolin (in pink). Reduction of nucleolin accounted for approx. 35% loss in binding affinity. These results confirm that the radioligand is bound to tumour endothelial cells via the nucleolin receptor (NR).


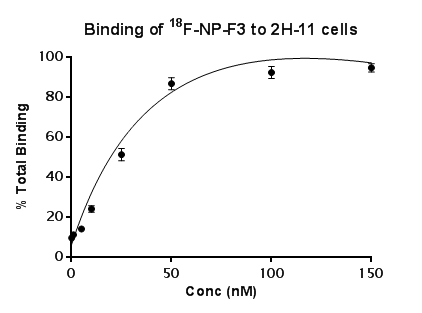


**Supplementary information to Figure. 5a.** **Saturation binding curve of ^18^F-NP-F3 on 2H-11 cells.** ^18^F-NP-F3 was added to 2H-11 cells in varying concentrations. The kd value was found to be 49 nM for 2H-11 cells, indicating that the compound retained specific affinity for the NR despite peptide modifications.
